# Use of Radon and CO_2_ for the Identification and Analysis of Short-Term Fluctuations in the Ventilation of the Polychrome Room Inside the Altamira Cave

**DOI:** 10.3390/ijerph19063662

**Published:** 2022-03-19

**Authors:** Carlos Sainz, Julia Fábrega, Daniel Rábago, Santiago Celaya, Alicia Fernandez, Ismael Fuente, Enrique Fernandez, Jorge Quindos, Jose Luis Arteche, Luis Quindos

**Affiliations:** 1 Radon Group, University of Cantabria, 39011 Santander, Spain; carlos.sainz@unican.es (C.S.); julia.fabrega@alumnos.unican.es (J.F.); santiago.celaya@unican.es (S.C.); alicia.fernandezv@unican.es (A.F.); ismael.fuente@unican.es (I.F.); enrique.fernandez@unican.es (E.F.); jorge.quindos@unican.es (J.Q.); luis.quindos@unican.es (L.Q.); 2 The Cantabrian International Institute for Prehistoric Research (IIIPC), 39005 Santander, Spain; 3 Spanish Meteorological Agency (AEMET), 39012 Santander, Spain; jartecheg@aemet.es

**Keywords:** radon, CO_2_, tracer, rock art, cave

## Abstract

A study is presented on rapid episodes of air exchange in the Polychrome Room of the Altamira Cave (Cantabria, Spain) using continuous monitoring of radon and CO_2_ tracer gases, as well as environmental parameters such as internal and external air temperature. For this, criteria have been developed to carry out an inventory of these types of events during the 2015–2020 period. Most of the degassing-recharging events occur over several hours or days, especially during spring and autumn. This means that the room can be significantly ventilated during these short periods of time, posing an exchange of energy and matter with potential impact in the preservation of the rock art present inside. In addition, the hypothesis that temperature gradients between the internal and external atmosphere is one of the main factors that induces degassing has been tested. To this end, correlation analysis has been carried out between the different magnitudes involved in this study, such as radon and CO_2_ concentrations, and air temperature gradients. A total of 37 degassing-recharging events have been analyzed for the 5 year studied period. The distribution of the duration of the events have been described, as well as that of the correlations between the degassing and recharge stages of each event, showing significant values of r coefficients for the correlation with temperature gradients between the internal and external atmosphere.

## 1. Introduction

Natural underground cavities are a recurrent subject of study for a variety of reasons. Their formation and morphology are both related to a wide range of geophysical and geochemical processes, the study of which provides valuable scientific information that allows us to understand, manage and effectively preserve the rock art contained in the caves. In the case of tourist caves, a management system based on observation and continuous analysis of environmental parameters is essential for their proper use and especially to correctly preserve the heritage contained within [1,2,3,4]

One of the key aspects in the environmental monitoring of caves is the study of atmospheric dynamics, particularly focused on characterising the degree of gas exchanged between the cavity and the external atmosphere. Cave ventilation influences both the internal air quality and the transport of physical, chemical and biological agents that can alter the preservation conditions of artistic manifestations and formations such as speleothems [5,6,7,8,9,10,11].

For this type of analysis, CO_2_ and radon (^222^Rn) gases, which are always present in higher concentrations in the underground environment than in the outside air, are fundamental as tracers. In spite of radon and CO_2_ sources and sinks being quite different, their temporal variations run almost in parallel. Even if the production rate is not quantified, continuously monitoring the concentrations of both gases serves a dual purpose. Firstly, these gases can pose a risk to human health depending on their concentration. In the case of carbon dioxide, its concentration in internal air also has a significant influence on the state of preservation of cave paintings and speleothems [12,13,14]. The second major area of interest in the study of these gases in caves is their utility as tracers of the air renovation of enclosures [15,16]. Time variations in the concentrations of these gases are used to construct models that describe and quantify the degree of connection between internal and external air. Generally, these models result in the identification of seasonal air exchange cycles associated with external thermal variations, whose intensity can vary significantly from cave to cave [17]. It is common to find in literature, cycles of indoor gas accumulation associated with a higher degree of insulation from the external atmosphere during the spring-summer period, followed by cycles of degassing during the autumn-winter period. However, this is not a standard rule, as the distribution of such cycles may vary depending on the morphology of the cave [18,19,20,21,22,23]. 

The Altamira cave is one of the most extensively studied cases in the world; time series of the main environmental parameters over a period of more than thirty years are currently available. The air exchanges of the cave and particularly of the Polychrome Room, where most of the rock art is located, has been described in detail since the 1980s. Consequently, annual cycles of degassing and gaseous recharging, which occur between spring-summer and autumn-winter, respectively, have been clearly identified. The relationship between these dynamics and the thermal gradients between the inside and outside of the cave has also been clearly established. The seasonal relationship between the concentration of gases inside the Polychrome room and the air temperature gradient described in this study confirms the trend observed in the Altamira cave since the first systematic studies started in the 1980s. It is remarkable that this relationship is opposite that usually described in the literature on other caves, and that the cause of such differences may be related to the morphology of each cave. Altamira is a shallow karstic cave and the analysis of the differences that this fact supposes on the gas dynamics with respect to other morphologically different caves will be the object of the continuation of this study [13,17,24].

Nonetheless, in addition to the aforementioned gas dynamics, there may exist degassing/recharging episodes of a much shorter period, of the order of hours/days, which until now have only been described on an ad hoc basis [16,25]. This is a relatively frequent phenomenon at certain period of the year and its relevance should be considered for the preservation of the rock art. This study furthermore presents an examination of correlations between Rn, CO_2_ concentrations and internal/external thermal gradients and preliminarily addresses the existence of time lags between the variables involved, which will be the subject of more detailed studies in future. 

The aim of this paper is to study the factors that cause air exchanges in the Polychrome Room during short periods of time as it may have an impact on the transport of materials from outside, which may influence the preservation conditions of the rock art contained in this room. For this purpose, an inventory was made of events where large degassing events occur during several days in the 2015–2020 period. Most of these short degassing and recharging processes, where the cave undergoes strong air exchanges, take place during spring and autumn.

## 2. Materials and Methods

### 2.1. Instrumentation

In this study, the database containing the measurement of environmental parameters monitored continuously in the cave was used. There are monitoring stations located at different points in the cave that measure the temperature at different heights, the atmospheric pressure, the CO_2_ concentration and the relative humidity. The variables are measured every minute in the Polychrome Room and every 15 min in the other locations. The picture of each sampling station, its location and additional information can be found in [16].

**Figure 1:**
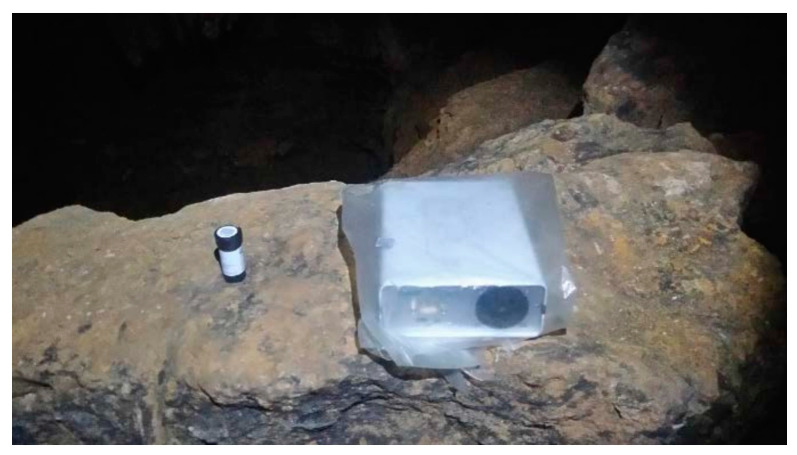
Alpha track detector CR-39 (on the left) and Radon Scout monitor in a plastic bag (on the right).

The radon concentration is recorded continuously every hour using Radon Scout (Sarad GmbH, Dresden, Germany) monitors in the Hall, the Polychrome and the Well Room. Due to possible drifts in the operation of this equipment caused by the influence of high humidity in the cave, the monitors are replaced every 14 days and are placed in waterproof plastic bags with a high permeability to radon. As an additional quality control measure, the time-integrated radon concentration is measured over the same exposure period with passive CR-39 detectors (Radosys, Budapest, Hungary). These devices and the quality control of the measurements have been previously described in [24]. The two radon measurement devices used in this study can be seen in Figure 1.

### 2.2. Time Series of Environmental Parameters Used

The provided database contains a range of experimental measurements of environmental parameters of the Polychrome Room and the exterior of the cave taken from 1 January 2015 to 31 December 2020. The parameters used are as follows:

- Radon concentration of the Polychrome Room *C_Rn_* (Bq/m^3^).
- CO_2_ concentration of the Polychrome Room *C_CO_*_2_ (ppm).
- Mid-height temperature of the Polychrome Room at 80 cm *T*_poly_ (°C).
- Mid-height temperature of the exterior of the cave at 80 cm *T*_ext_ (°C).

The time frequency of each of the parameters used is given every 15 min except for radon concentration which is given hourly. 

**Figure 2:**
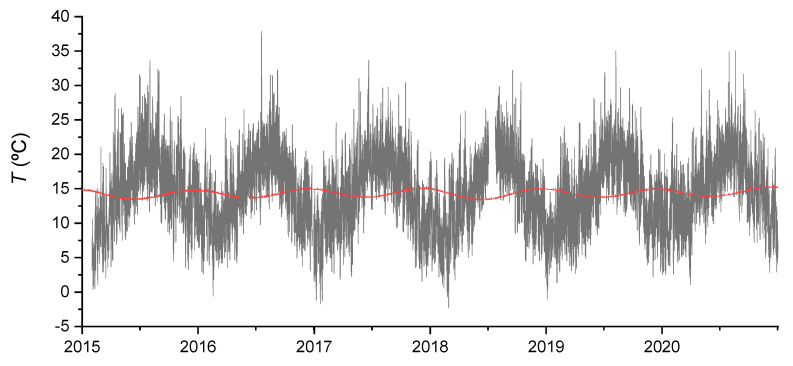
Temperature time series of the exterior of the cave (black), Polychrome Room (red) from 2015 to 2020.

Figure 2 shows the time series of temperatures for the Polychrome Room and the exterior zone of the cave from 2015 to 2020 where the different amplitudes and the time series lag can be seen. 

**Figure 3:**
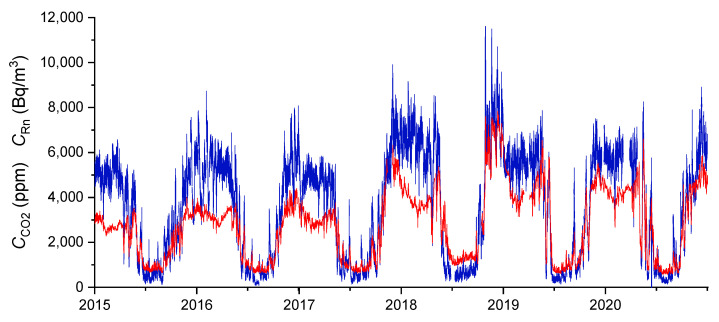
Time series of radon concentration (blue) and of CO_2_ (red) in the Polychrome Room from 2015 to 2020.

The CO_2_ and radon concentration time series show well-defined seasonal oscillations. The highest concentrations of both gases occur between November and April and the lowest concentrations between June and October. Figure 3 shows the time series of the Rn and CO_2_ concentration for the Polychrome Room. By studying this behaviour, it can be seen how the evolution of both gases occurs in parallel. 

Although the annual variation of the temperature gradient between the external and internal atmosphere shows well defined periodic oscillations, a short period of degassing and recharging episodes was detected. These events consist of a large mass of moving air released from the cave for a few hours or days.

### 2.3. Degassing and Recharging Identification Criteria

The identification of degassing and recharging is obtained from the sudden decrease and increase in radon and CO_2_ concentrations. For this purpose, two criteria have been adopted: the first is temporal, meaning a short-term event was defined as one that occurs within a maximum of twelve days; the second is that of relative variation, which determines that the percentage decrease or increase of gases should be over 40% in the established period of time. 

### 2.4. Correlations

Once the degassing and recharging events were selected, an analysis of correlations between the gas concentration and the temperature gradient between the outside of the cave and the Polychrome Room was carried out: ∆*T* = *T_ext_* − *T_poly_*. The temperature gradient has been selected instead of the density gradient because the temperature is the variable directly measured in the experiment. In the research of [16], it was shown that the correlation between temperature gradient and gases is sufficiently high and significant to consider this gradient as an indicator and cause of air movements. This makes it easier to identify and predict these events depending on the internal and external temperature variations. In addition, the correlation between gas concentrations has been analysed in order to compare their behaviour and try to identify if there is a time lag between them. 

The method implemented to determine the lag between two time series is based on the cross-correlation technique [26]. It involves performing a known time shift *h* of a series *x_t_* with respect to another series *y_t_* and calculating the Pearson correlation coefficient *r*_xy_. Performed repeatedly, this procedure yields a set of correlation coefficients with their corresponding significance for each of the shifts. The shift with the highest correlation coefficient determines the lag. 

The study of correlations between the temperature gradient and gas concentrations has been carried out individually both for degassing and recharging events, as well as for the whole period comprising both processes. For the correlations between *C_Rn_* and ∆*T*, and between *C_Rn_* and *C_CO_*_2_, hourly measured values have been used because the radon concentration has been measured hourly. In contrast, for the correlations between *C_CO_*_2_ and ∆*T*, all corresponding values have been selected, as 15-min values were available.

## 3. Results

### 3.1. Event Inventory

**Figure 4:**
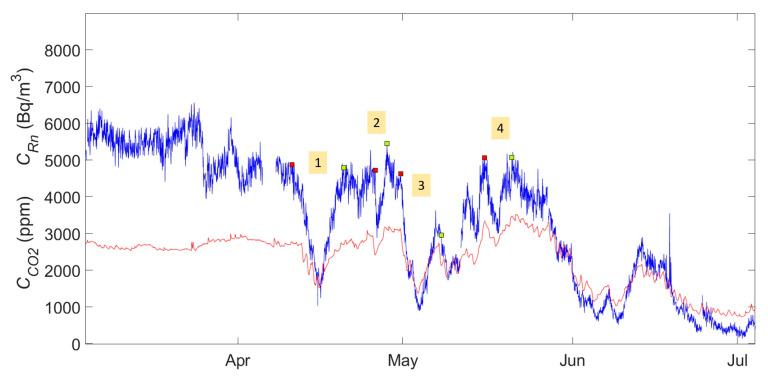
Degassing and recharging events identified in the first half of 2015 labeled with number 1, 2, 3 and 4 for radon (blue) and CO_2_ (red). Red markers show the start of the degassing, and the green ones show the end of recharge.

**Figure 5:**
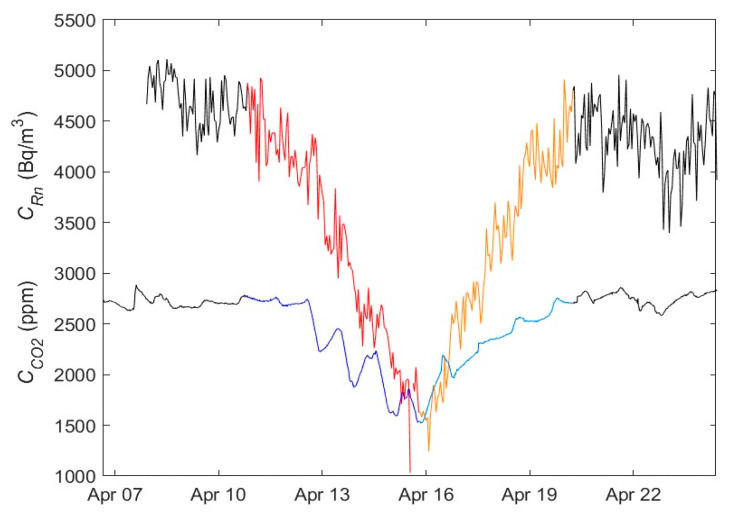
Variation in the gas concentration of event 1 in April 2015, which is shown in Figure 4. The radon degassing is shown (in red), the recharging (orange) and the CO_2_ degassing (dark blue) and the recharging (light blue).

**Figure 6:**
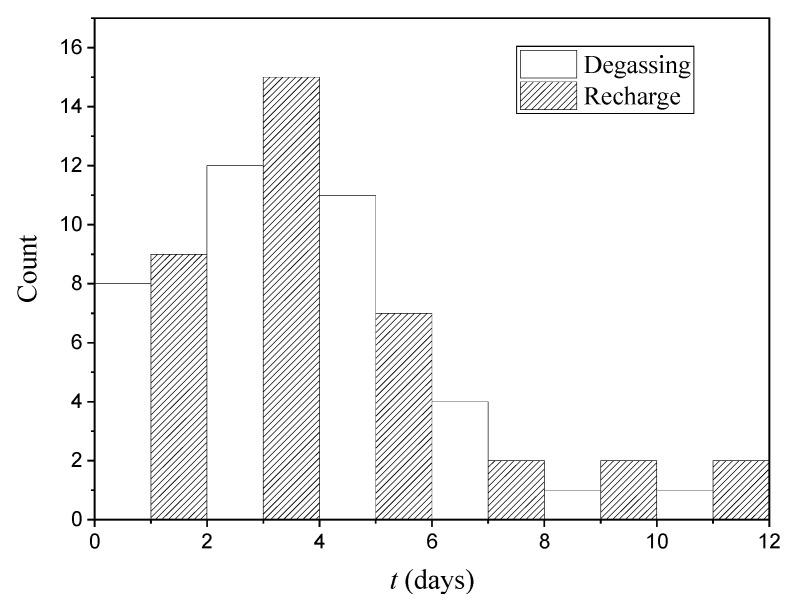
Distribution of duration of degassing and recharge events. Dates of every event are shown in Supplementary Materials.

Based on the established criteria, degassing and recharging events have been identified and compiled for the 2015–2020 period. As an example, Figure 4 shows the evolution of the radon and CO_2_ concentrations. In the first half of 2015, four first events were identified and labeled with the numbers 1, 2, 3 and 4. The graphical representation of each of the events can be found in the Supplementary Materials. A detailed graph of event number one is shown in Figure 5. Figure 6 shows the distribution of duration of degassing and recharge events measured from the variations of the concentration of both gases, CO_2_ and radon. 

### 3.2. CO_2_ and Rn Correlations

The events studied show that the degassing and recharging processes of the studied gases have an earlier temporary effect in the case of CO_2_. It can be seen that the decrease in CO_2_ concentration starts earlier than the decrease of radon. The same applies for the minimum value as the transition point between degassing and recharging occurs earlier (see Figure 4). This happens systematically to a greater or lesser extent.

From the application of the maximum cross-correlation method, the time lag between Rn and CO_2_ has been obtained for each of the events. An average result of 5.3 ± 2.7 h was obtained.

The lag between the behaviour of Rn and CO_2_ does not make physical sense as it represents the main driven force for the convective transport of air masses containing CO_2_ and radon. Therefore, the concentration of both gases should increase and decrease at the same time. This means that air masses move due to temperature and pressure variations, although diffusion also occurs simultaneously to a much lesser extent. It can therefore be hypothesized that the obtained lag is due to the measuring conditions of the Radon Scout devices, which are placed in plastic bags in order to protect their electronic parts from the humidity. Consequently, the bag acts as an Rn retarder interphase, meaning that it takes radon a few hours to reach the actual concentration of the cave when it diffuses through the bag. In this case, the movement of the Rn is not caused by the pressure and temperature gradients between the bag and the cave, but the difference between concentrations. This fact has been confirmed by the radon chamber of LaRUC [27], which allows to simulate variations similar to those produced in the cave and quantify the lags between the actual radon concentration and the one obtained from the device contained in the plastic bag.

### 3.3. Correlations between Gases and Temperature Gradients

**Figure 7:**
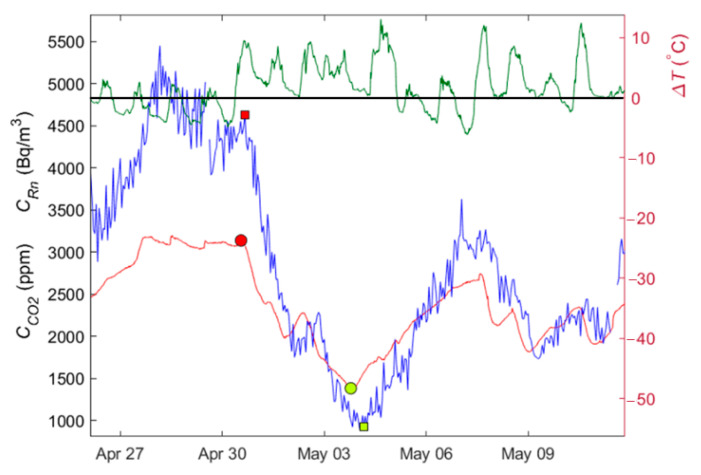
Radon concentration variation (blue) and CO_2_ (red) for event number 3 in May 2015. The temperature gradient is shown (green) and the 0 °C is marked with a black line. Red markers show the start of the degassing, and the green ones show the end; a square is used for Rn and a circle for CO_2_.

The temperature gradient, CO_2_, and radon concentrations have been represented in individual graphs for each event analysed. In this case, a lag is also observed in the concentration of the gases and the temperature gradient. Figure 7 shows how the increase in the gradient and, more specifically, when it becomes positive, does not instantly start the process of CO_2_ degassing. This is to be expected because the gradient change cannot cause an instantaneous movement of the air masses and, consequently, an instantaneous change of the concentration of gases. Therefore, the study of correlations between the concentration of gases and the temperature gradient has been made by looking for the maximal correlation, i.e., moving the time series of each of the gases with respect to the gradient. A high variability is observed in the lags of the analysed events to obtain a maximal correlation. This may be due to the high variability in the conditions of the karst system, the different periods analysed, the permeability of the terrain, etc., which will be the subject of future research. It should be noted that the time lag considered in all cases is in the same order of magnitude of event duration (hours). 

**Figure 8:**
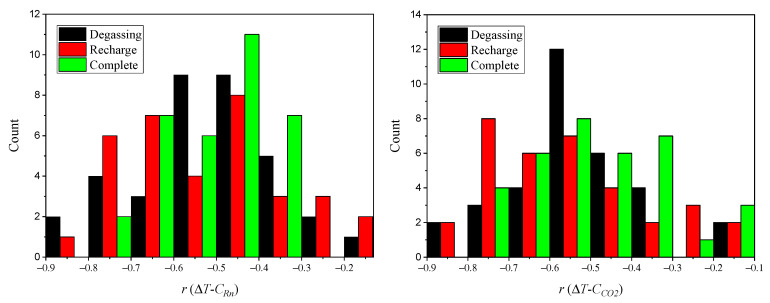
Distribution of maximum correlation coefficient *r* between the temperature gradient Δ*T* and the concentration of gases, Rn and CO_2_, in degassing, recharging events or in complete periods.

Figure 8 shows the distribution of the maximum correlation coefficient between the temperature gradient and the concentration of gases in degassing, recharging events or in complete periods. They are divided into the periods of degassing, recharging and complete event. In all cases, the correlation between the temperature gradient and the gases concentration is negative, i.e., when the outside temperature is higher than the one corresponding to the cave, the system gets degassed. Likewise, when the outside temperature is lower than the one inside the cave, the system gets insulated, and the gases accumulate. For events 7 (recharging), 8 and 9, correlations were not performed because of the lack of temperature data in this period due to technical issue with datalogger. 

The average and the standard deviation were obtained for the total set of events for each of the stages. In the case of degassing, an average correlation coefficient of −0.53 between the temperature gradient and radon is obtained and −0.55 for the corresponding CO_2_ case. The standard deviations show that 68% of coefficients are between −0.68 and 0.38 for radon and between −0.73 and −0.37 for CO_2_. This shows that the dispersion of the correlations is relatively small. 

In the recharging event, the same is obtained for the correlation between the temperature gradient and radon. As for the correlation between the gradient and CO_2_, the coefficient is −0.56 and the standard deviation is 0.19, i.e., 68% of the values are between −0.75 and −0.37. 

For the complete events, the average correlation coefficients for both gases are −0.49. 68% of the coefficients are between−0.63 and 0.35 for radon and between −0.66 and −0.32 for CO_2_.

## 4. Conclusions

We have confirmed, as showed in previous studies, that air temperature/density gradients between the inner and outer atmosphere are the driving forces which explain temporal variations in the indoor concentrations of gases in Altamira cave. The accumulation period happens during wintertime in this cave. On the other hand, we found that the trace gas concentrations reach their minimum annual values during summertime. This is a fact observed systematically during the last 30 years of continuous observations carried out by different research groups. The explanation for this behaviour can be related to the cave´s morphology by taking into account that Altamira is a shallow and karstic formation. The movement of air masses with different temperature is caused by gravitational forces, which are responsible for different seasonal trends depending on the depth of the cave room studied, and also on the degree of connection of the rocks with the outdoor atmosphere. Comparison of seasonal gas concentration trends between caves with different depth, degree of isolation and morphology will be showed further studies.

An inventory of short-period degassing events was made from 2015 and 2020, 37 of which were found for the established criteria. More events than the ones analysed occurred and there were more than expected considering the general dynamics of the time series over the six years. These events have been studied to determine if the temperature gradient between the exterior and the interior of the cave is a determining factor which causes these events. Thus, the concentrations of tracer gases such as CO_2_ and radon, are used to indicate if the cave is insulated or, on the contrary, ventilated. 

The Polychrome Room presents short period gaseous exchanges more frequently in seasons when the greatest thermal contrasts between day and night occur. One of these seasons is spring, when the temperature gradient increases. This causes water evaporation in the soil/rock layer, which blocks the cracks that surround the cave, allowing the exchange of air masses between the cave and the exterior. The other season is autumn, when the temperature gradient starts decreasing and rainwater starts to accumulate in the cracks of the karstic system. 

The correlation coefficients found between the concentration of gases and the exterior-interior temperature gradient are significant and indicate frequent values in the study of complex processes such as this one. The observed correlations confirm that the temperature gradient between the exterior and the interior of the cave is a key factor for the large degassing events through karstified rocks. Similarly, and because the temperature in the Polychrome Room remains approximately constant during each of these events, the exterior temperature variation could be a good indicator and predictor of the above-mentioned events. 

A time lag between the temperature gradient and the concentration of gases in the cave has been observed, showing that the temperature gradient change produces a degassing/recharge event in a progressive manner. A better knowledge of the latter will allow the refinement of the estimation of the correlation coefficients showed in this study. 

The detailed knowledge of the intensity and frequency of the degassing recharge episodes studied in this research has, from our point of view, relevance regarding the preservation of rock art from two different perspectives. On the one hand, the entrance of outdoor air could bring biological related contamination, which have been one of the most important hazards detected in caves containing rock art. On the other hand, the preventive conservation of rock art contained in Altamira cave includes a complex system involving protocols for controlling different types of risks. One of these protocols is related to the control of indoor air CO_2_ concentration and the anthropic influence on that variable. The identification and understanding of the analyzed degassing/recharge episodes, which occurs naturally with independence of human presence inside the cave, allows us to discriminate more clearly the real anthropic influence, avoiding false alarms due to a wrong attribution of sudden CO_2_ concentration changes.

## Data Availability

Not applicable.

## Supplementary Materials

The following supporting information can be downloaded at: https://www.mdpi.com/article/10.3390/ijerph19063662/s1, Figure S1: Graphs of every degasification and recharge events. Table S1. Time record, start and end of the n selected degassing-recharging events considering CO_2_ as a reference. Table S2. Maximum correlation coefficient between the temperature gradient and the concentration of gases in degassing, recharging events or in complete periods. The average value and the standard deviation (SD) are reported.
