# Supplementary material for: Use of Radon and CO2 for the Identification and Analysis of Short-Term Fluctuations in the Ventilation of the Polychrome Room Inside the Altamira Cave
Source: Int J Environ Res Public Health. 2022 Mar 19;19(6):3662. doi: 10.3390/ijerph19063662 (PMC8955978; doi:10.3390/ijerph19063662)
Supplement: Supplementary file 1 [file ijerph-19-03662-s001.zip › ijerph-1592538-supplementary.pdf]

Table S1. Time record, start and end of the *n* selected degassing-recharging events considering CO_2 as a reference.

n	Start of degassing	End of discharge/start of recharging	End of recharging
1	10-Apr-2015 20 h	15-Apr-2015 19 h	20-Apr-2015 06 h
2	26-Apr-2015 00 h	26-Apr-2015 08 h	28-Apr-2015 04 h
3	30-Apr-2015 16 h	04-May-2015 04 h	08-May-2015 01 h
4	15-May-2015 23 h	18-May-2015 07 h	20-May-2015 22 h
5	04-Oct-2015 20 h	06-Oct-2015 14 h	10-Oct-2015 13 h
6	10-Oct-2015 15 h	12-Oct-2015 21 h	17-Oct-2015 02 h
7	17-Oct-2015 22 h	19-Oct-2015 20 h	23-Oct-2015 20 h
8	20-May-2016 07 h	21-May-2016 23 h	23-May-2016 20 h
9	23-May-2016 20 h	28-May-2016 09 h	01-Jun-2016 22 h
10	18-Jun-2016 22 h	23-Jun-2016 20 h	27-Jun-2016 14 h
11	15-Oct-2016 09 h	19-Oct-2016 05 h	22-Oct-2016 22 h
12	22-Oct-2016 22 h	27-Oct-2016 16 h	29-Oct-2016 00 h
13	25-Mar-2017 08 h	31-Mar-2017 05 h	05-Apr-2017 17 h
14	05-Apr-2017 17 h	09-Apr-2017 21 h	11-Apr-2017 15 h
15	20-Apr-2017 20 h	24-Apr-2017 02 h	28-Apr-2017 09 h
16	14-May-2017 00 h	17-May-2017 03 h	20-May-2017 01 h
17	12-Oct-2017 07 h	16-Oct-2017 20 h	19-Oct-2017 20 h
18	25-Oct-2017 05 h	28-Oct-2017 16 h	01-Nov-2017 13 h
19	01-Nov-2017 13 h	04-Nov-2017 02 h	11-Nov-2017 01 h
20	11-Apr-2018 18 h	22-Apr-2018 10 h	03-May-2018 16 h
21	03-May-2018 16 h	08-May-2018 07 h	10-May-2018 17 h
22	12-Apr-2019 06 h	15-Apr-2019 08 h	19-Apr-2019 11 h
23	22-Apr-2019 18 h	23-Apr-2019 10 h	24-Apr-2019 10 h
24	06-May-2019 19 h	09-May-2019 01 h	10-May-2019 08 h
25	14-May-2019 04 h	16-May-2019 10 h	19-May-2019 22 h
26	22-May-2019 10 h	23-May-2019 22 h	25-May-2019 10 h
27	29-May-2019 09 h	02-Jun-2019 21 h	06-Jun-2019 11 h
28	14-Jun-2019 15 h	20-Jun-2019 22 h	22-Jun-2019 19 h
29	11-Sep-2019 13 h	19-Sep-2019 12 h	11-Oct-2019 14 h
30	23-Oct-2019 21 h	02-Nov-2019 07 h	10-Nov-2019 13 h
31	04-Apr-2020 04 h	05-Apr-2020 18 h	07-Apr-2020 14 h
32	09-Apr-2020 12 h	11-Apr-2020 01 h	14-Apr-2020 16 h
33	14-Apr-2020 16 h	18-Apr-2020 09 h	22-Apr-2020 04 h
34	15-May-2020 23 h	23-May-2020 03 h	25-May-2020 08 h
35	25-May-2020 08 h	30-May-2020 01 h	05-Jun-2020 03 h
36	30-Agu-2020 11 h	05-Sep-2020 19 h	09-Sep-2020 18 h
37	05-Oct-2020 13 h	09-Oct-2020 14 h	17-Oct-2020 17 h

Table S2. Maximum correlation coefficient between the temperature gradient and the concentration of gases in degassing, recharging events or in complete periods. The average value and the standard deviation (*SD*) are reported.

	**Degassing**		**Recharging**		**Complete**	
n	r (ΔT- C_{Rn})	r (ΔT- C_{CO2})	r (ΔT- C_{Rn})	r (ΔT- C_{CO2})	r (ΔT- C_{Rn})	r (ΔT- C_{CO2})
1	-0.79	-0.87	-0.65	-0.73	-0.66	-0.73
2	-0.81	-0.91	-0.52	-0.58	-0.51	-0.70
3	-0.49	-0.52	-0.60	-0.60	-0.51	-0.54
4	-0.44	-0.60	-0.35	-0.44	-0.41	-0.51
5	-0.63	-0.66	-0.36	-0.15	-0.32	-0.32
6	-0.45	-0.53	-0.68	-0.72	-0.66	-0.68
7	-0.44	-0.34			-0.44	-0.34
8						
9						
10	-0.51	-0.56	-0.45	-0.67	-0.37	-0.38
11	-0.39	-0.47	-0.68	-0.68	-0.64	-0.62
12	-0.20	-0.19	-0.66	-0.69	-0.14	-0.15
13	-0.61	-0.54	-0.53	-0.53	-0.51	-0.51
14	-0.54	-0.58	-0.28	-0.34	-0.50	-0.50
15	-0.30	-0.12	-0.67	-0.64	-0.40	-0.20
16	-0.38	-0.44	-0.74	-0.80	-0.53	-0.53
17	-0.81	-0.87	-0.79	-0.81	-0.54	-0.62
18	-0.26	-0.45	-0.49	-0.57	-0.43	-0.55
19	-0.40	-0.47	-0.44	-0.58	-0.49	-0.63
20	-0.73	-0.74	-0.73	-0.79	-0.72	-0.74
21	-0.59	-0.61	-0.50	-0.56	-0.53	-0.58
22	-0.71	-0.73	-0.21	-0.11	-0.36	-0.38
23	-0.79	-0.92	-0.78	-0.74	-0.66	-0.72
24	-0.50	-0.52	-0.31	-0.29	-0.44	-0.40
25	-0.31	-0.51	-0.61	-0.56	-0.40	-0.51
26	-0.40	-0.38	-0.55	-0.62	-0.41	-0.37
27	-0.58	-0.59	-0.45	-0.50	-0.40	-0.30
28	-0.51	-0.46	-0.18	-0.38	-0.44	-0.38
29	-0.53	-0.55	-0.42	-0.44	-0.34	-0.45
30	-0.58	-0.56	-0.77	-0.78	-0.69	-0.60
31	-0.69	-0.78	-0.83	-0.82	-0.72	-0.78
32	-0.47	-0.37	-0.73	-0.72	-0.63	-0.50
33	-0.48	-0.31	-0.14	-0.25	-0.19	-0.11
34	-0.50	-0.49	-0.42	-0.41	-0.46	-0.42
35	-0.54	-0.62	-0.48	-0.64	-0.48	-0.44
36	-0.56	-0.64	-0.24	-0.28	-0.42	-0.47
37	-0.47	-0.52	-0.69	-0.73	-0.63	-0.66
Average	-0.53	-0.55	-0.53	-0.56	-0.49	-0.49
SD	0.15	0.18	0.19	0.19	0.14	0.17

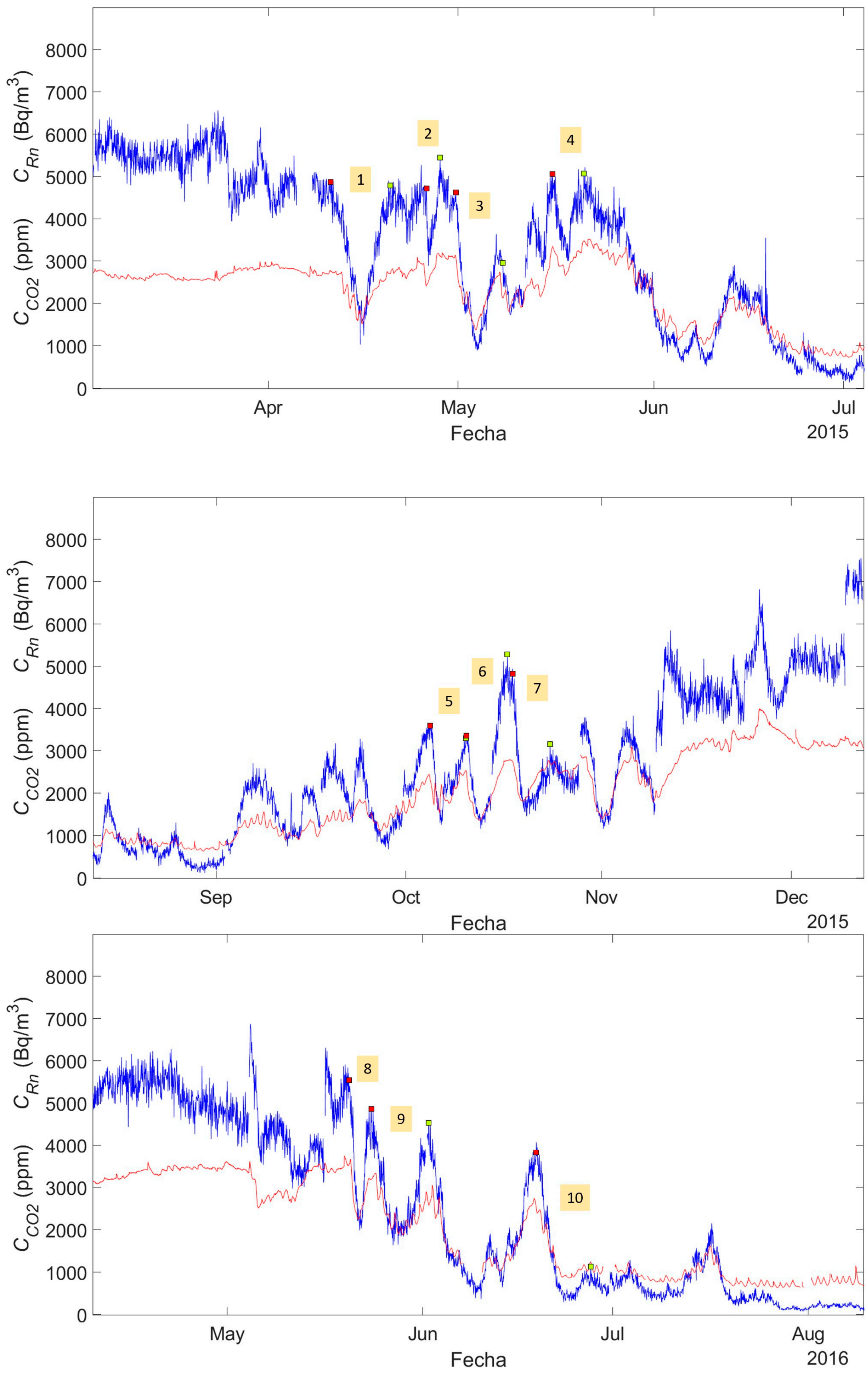

C_{Rn} (Bq/m^3)
C_{CO2} (ppm)
0
1000
2000
3000
4000
5000
6000
7000
8000
1
2
3
4
Apr
May
Jun
Jul
Fecha
2015
5
6
7
Sep
Oct
Nov
Dec
Fecha
2015
8
9
10
May
Jun
Jul
Aug
Fecha
2016

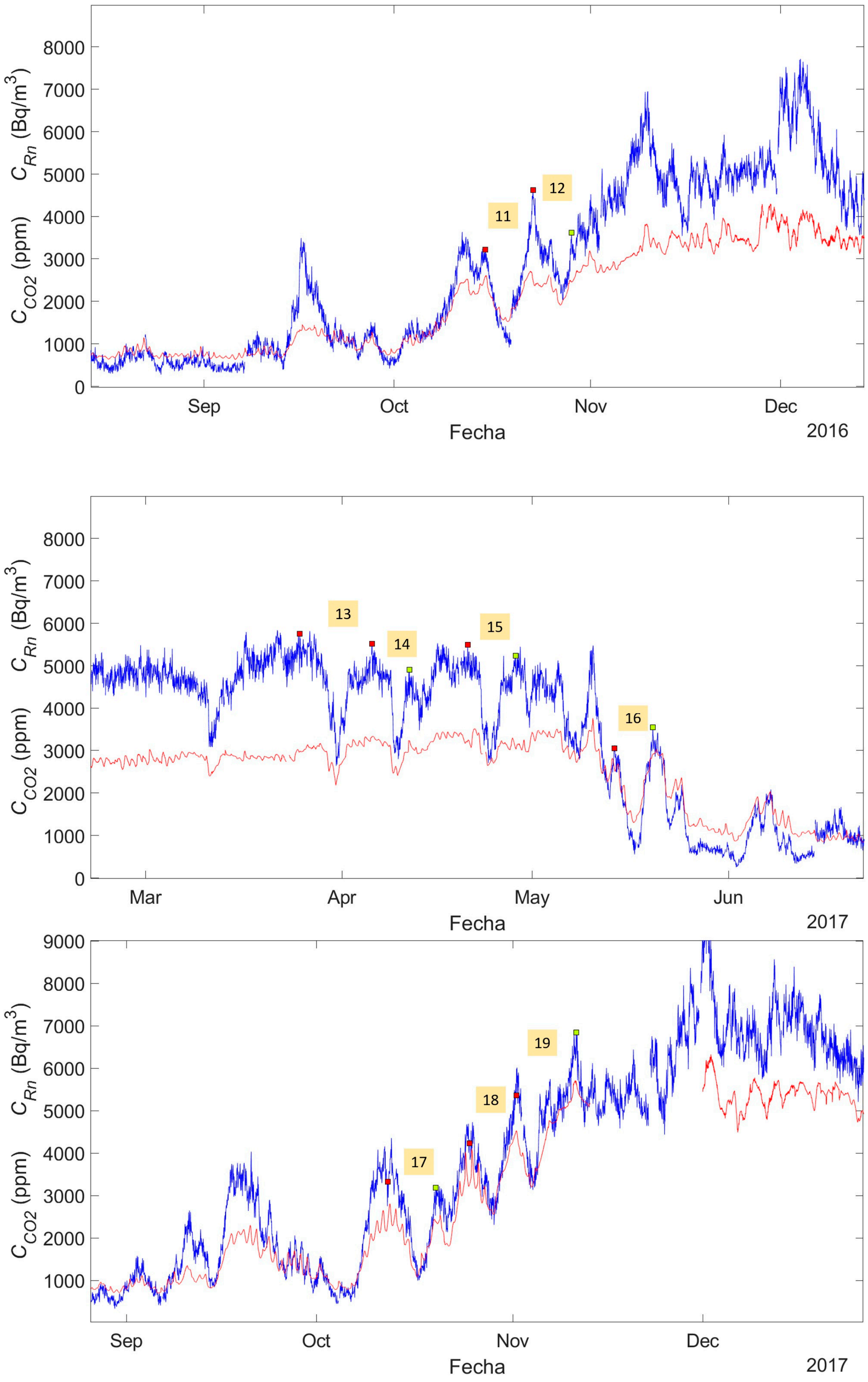

C_{CO2} (ppm) C_{Rn} (Bq/m^3)
0
1000
2000
3000
4000
5000
6000
7000
8000
11
12
Sep
Oct
Nov
Dec
Fecha
2016
C_{CO2} (ppm) C_{Rn} (Bq/m^3)
0
1000
2000
3000
4000
5000
6000
7000
8000
13
14
15
16
Mar
Apr
May
Jun
Fecha
2017
C_{CO2} (ppm) C_{Rn} (Bq/m^3)
1000
2000
3000
4000
5000
6000
7000
8000
9000
17
18
19
Sep
Oct
Nov
Dec
Fecha
2017

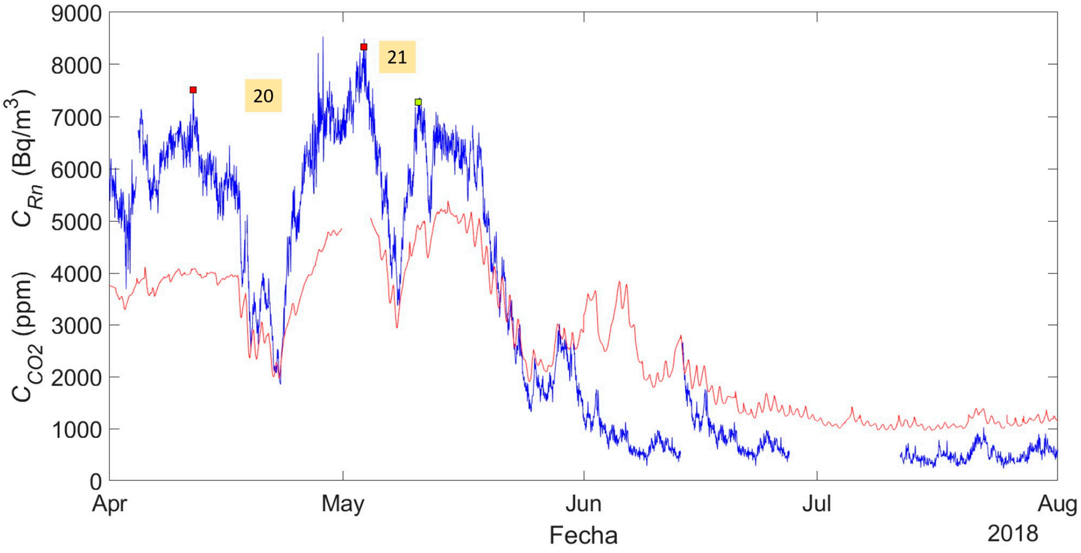

9000
8000
7000
6000
5000
4000
3000
2000
1000
0
C_{Rn} (Bq/m^3)
C_{CO2} (ppm)
20
21
Apr
May
Jun
Jul
Aug
Fecha
2018

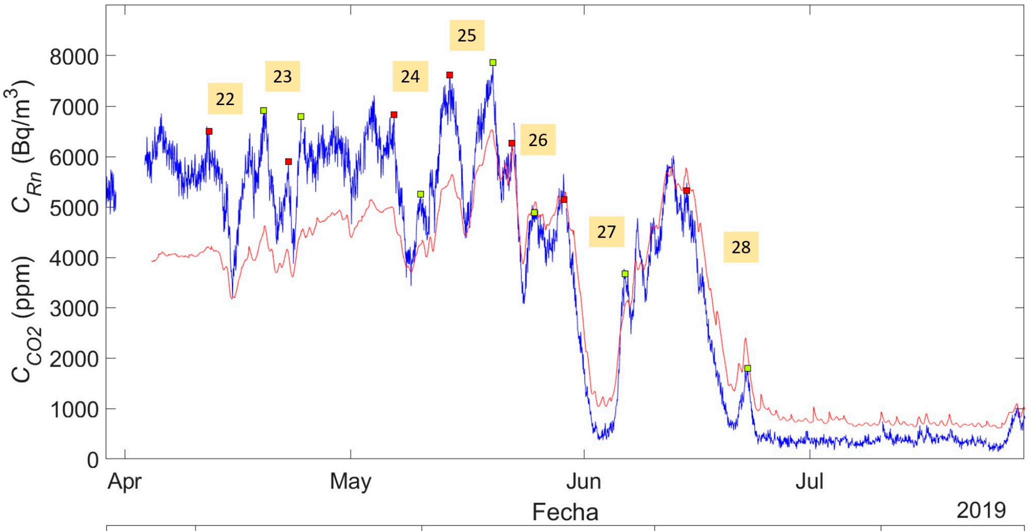

8000
7000
6000
5000
4000
3000
2000
1000
0
C_{Rn} (Bq/m^3)
C_{CO2} (ppm)
22
23
24
25
26
27
28
Apr
May
Jun
Jul
Fecha
2019

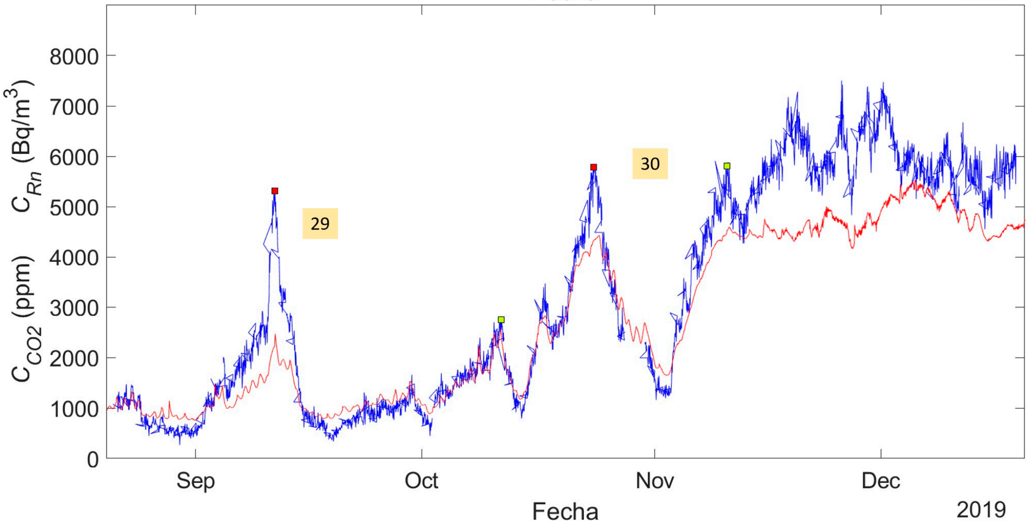

8000
7000
6000
5000
4000
3000
2000
1000
0
C_{Rn} (Bq/m^3)
C_{CO2} (ppm)
29
30
Sep
Oct
Nov
Dec
Fecha
2019

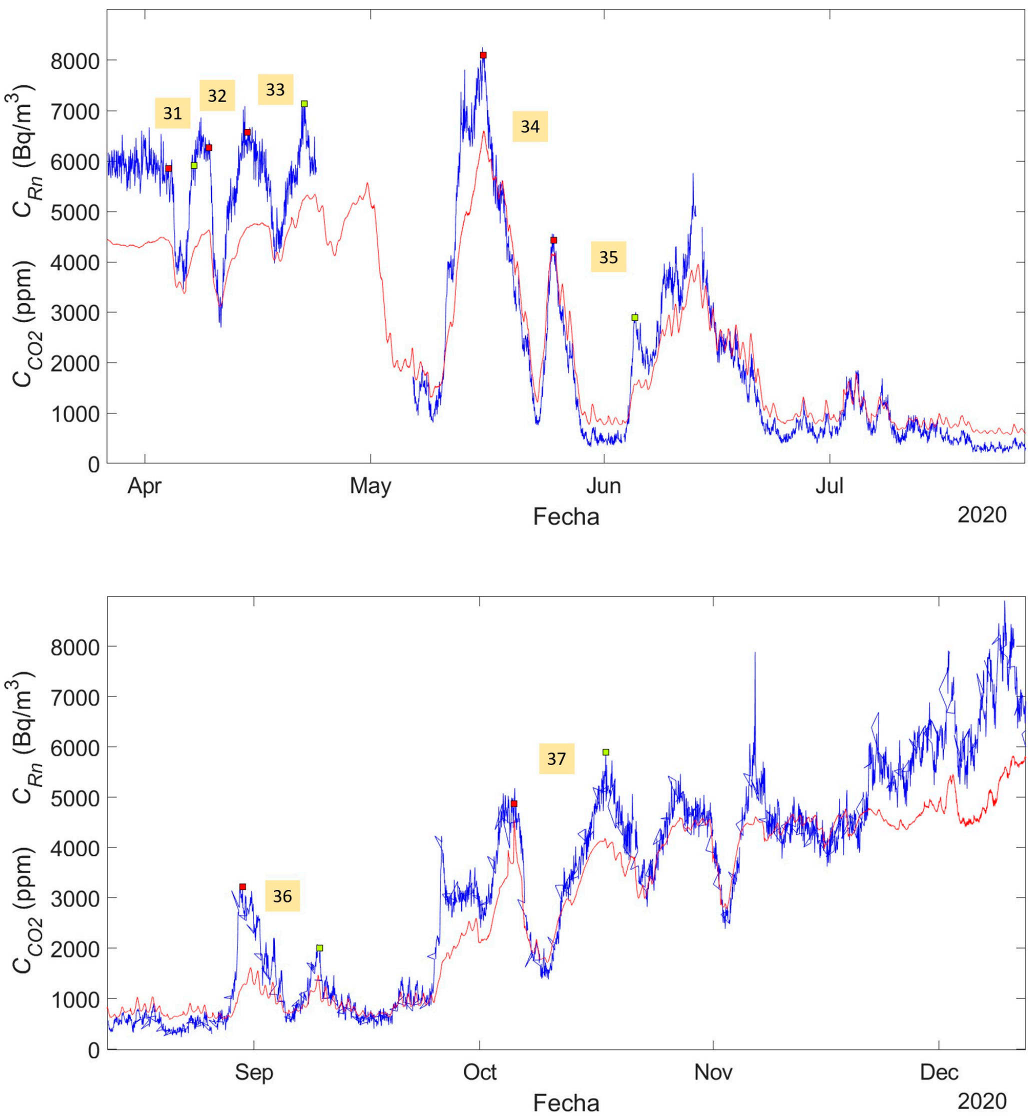

Figure S1. Graphs of every degasification and recharge events.
